# IL11 Activates Pancreatic Stellate Cells and Causes Pancreatic Inflammation, Fibrosis and Atrophy in a Mouse Model of Pancreatitis

**DOI:** 10.3390/ijms23073549

**Published:** 2022-03-24

**Authors:** Benjamin Ng, Sivakumar Viswanathan, Anissa A. Widjaja, Wei-Wen Lim, Shamini G. Shekeran, Joyce Wei Ting Goh, Jessie Tan, Fathima Kuthubudeen, Sze Yun Lim, Chen Xie, Sebastian Schafer, Eleonora Adami, Stuart A. Cook

**Affiliations:** 1 National Heart Research Institute Singapore, National Heart Centre Singapore, Singapore 169609, Singapore; lim.wei.wen@nhcs.com.sg (W.-W.L.); jessie.tan@nhcs.com.sg (J.T.); xie.chen@nhcs.com.sg (C.X.); stuart.cook@duke-nus.edu.sg (S.A.C.); 2 Cardiovascular and Metabolic Disorders Program, Duke-National University of Singapore Medical School, Singapore 169857, Singapore; siva.viswanathan@duke-nus.edu.sg (S.V.); anissa.widjaja@duke-nus.edu.sg (A.A.W.); shamini_g@duke-nus.edu.sg (S.G.S.); joyce.goh@duke-nus.edu.sg (J.W.T.G.); fathima@duke-nus.edu.sg (F.K.); sy76@nus.edu.sg (S.Y.L.); sebastian@duke-nus.edu.sg (S.S.); 3 Cardiovascular and Metabolic Sciences, Max Delbrück Center for Molecular Medicine in the Helmholtz Association (MDC), 13125 Berlin, Germany; 4 MRC-London Institute of Medical Sciences, Hammersmith Hospital Campus, London W12 0NN, UK

**Keywords:** IL11, IL6, gp130, immune, ERK, therapy, cytokine

## Abstract

Interleukin-11 (IL11) is important for fibrosis and inflammation, but its role in the pancreas is unclear. In pancreatitis, fibrosis, inflammation and organ dysfunction are associated with pancreatic stellate cell (PSC)-to-myofibroblast transformation. Here, we show that IL11 stimulation of PSCs, which specifically express IL11RA in the pancreas, results in transient STAT3 phosphorylation, sustained ERK activation and PSC activation. In contrast, IL6 stimulation of PSCs caused sustained STAT3 phosphorylation but did not result in ERK activation or PSC transformation. Pancreatitis factors, including TGFβ, CTGF and PDGF, induced IL11 secretion from PSCs and a neutralising IL11RA antibody prevented PSC activation by these stimuli. This revealed an important ERK-dependent role for autocrine IL11 activity in PSCs. In mice, IL11 was increased in the pancreas after pancreatic duct ligation, and in humans, IL11 and IL11RA levels were elevated in chronic pancreatitis. Following pancreatic duct ligation, administration of anti-IL11RA to mice reduced pathologic (ERK, STAT, NF-κB) signalling, pancreatic atrophy, fibrosis and pro-inflammatory cytokine (TNFα, IL6 and IL1β) levels. This is the first description of IL11-mediated activation of PSCs, and the data suggest IL11 as a stromal therapeutic target in pancreatitis.

## 1. Introduction

Pancreatitis is an aetiologically heterogeneous fibro-inflammatory syndrome that is often precipitated by toxins, such as alcohol [1]. Pancreatitis results from damage to the exocrine parenchyma due to the premature intra-acinar activation of digestive enzymes. Recurrent acute pancreatitis can progress to chronic pancreatitis, which is characterised by inflammation and progressive fibrosis, ultimately leading to pancreatic atrophy and endocrine and exocrine pancreatic insufficiency [2,3,4]. Diagnosis of chronic pancreatitis typically occurs when the disease is established and the chance for improvement is poor, as no treatments currently exist to reverse the key drivers of disease progression, namely inflammation and fibrosis [5,6].

Pancreatic stellate cells (PSCs) have emerged as the predominant fibrogenic cell type in the injured pancreas [7,8,9,10]. In health, quiescent PSCs store vitamin A and maintain a normal turnover of extracellular matrix (ECM) proteins. In the diseased pancreas, PSCs in an activated state acquire a myofibroblast-like phenotype, characterised by overexpression of alpha smooth muscle actin (αSMA), excessive deposition of ECM components and secretion of pro-inflammatory factors [10]. Interestingly, PSCs are part of a wider retinoid-storing cellular network that includes hepatic stellate cells (HSCs) in the liver [11]. Despite organ-specific features, transcriptomic analyses comparing PSCs and HSCs show many similarities [12]. 

Interleukin-11 (IL11), a member of the IL6 family of cytokines, is implicated in a number of fibro-inflammatory diseases [13]. IL11 binds to its specific alpha receptor IL11RA, which is highly expressed on stromal cells (e.g., fibroblasts and HSCs) and signals via the gp130 receptor to activate ERK and, to a lesser degree and transiently, STAT3. Earlier studies have shown that IL11 is elevated in the serum of patients with severe pancreatitis [14,15,16]. Elevated IL6 has been associated with organ failure and severe pancreatitis [17] and may have a role in the activation of an immortalised PSC-like cell line [14]. The role of IL11 in PSC biology and in pancreatitis remains unclear. In a previous study in mice, Shimizu et al. showed that the recombinant human IL11 reduced the severity of pancreatic inflammation in caerulein-induced acute pancreatitis in mice [18]. 

Recently, we documented a previously unappreciated role for interleukin-11 (IL11) in the transformation of HSCs into myofibroblasts in the liver, a defining pathology in non-alcoholic steatohepatitis (NASH) [19]. Therapeutic inhibition of IL11 signalling with a neutralising antibody against IL11RA reversed liver inflammation and fibrosis in NASH. Given the similarities between the HSCs and PSCs and the established role of IL11 as a pro-fibrotic factor in several other tissues [13], we hypothesised that IL11 could be important for PSC activation and pancreatitis. 

Here, we explore the expression of IL11RA, IL6RA and gp130 in mouse and human PSCs and compare the effects of IL11 and IL6 treatment on PSC activation in vitro. We investigate whether autocrine IL11 signalling in PSCs is required for PSC activation downstream of various profibrotic cytokines implicated in pancreatitis. We test the therapeutic potential of a neutralising IL11RA antibody for the prevention of PSC activation in vitro and fibrosis in the pancreatic duct ligation (PDL) mouse model of pancreatitis in vivo. We also determine the expression of IL11 and IL11RA in pancreatic sections from patients. 

## 2. Results

### 2.1. IL11 Induces Pancreatic Stellate Cell Activation

**Figure 1:**
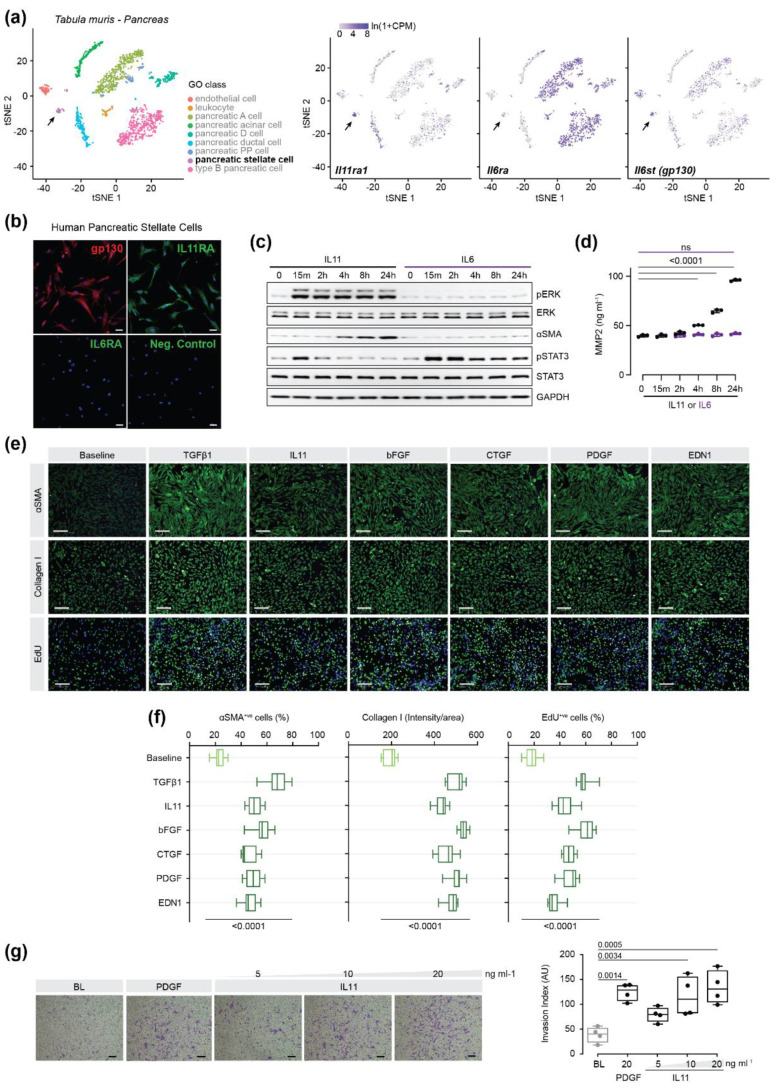
IL11 induces pancreatic stellate cell activation and invasion. (**a**) t-distributed stochastic neighbour embedding (tSNE) plots showing scRNA-seq expression of *Il11ra1*, *Il6ra* and *Il-6st* (*gp130*) in mouse pancreatic tissue. Cell clusters were identified using the Tabula Muris web tool (https://tabula-muris.ds.czbiohub.org/ (accessed on 21 February 2022)). [20]. Black arrows indicate pancreatic stellate cells (PSCs). (**b**) Representative immunostaining images of gp130, IL11RA, IL6RA in PSCs. Cells were counterstained with DAPI. Scale bars: 50 µm. (**c**) Western blot analysis of phosphorylated and total ERK and STAT3 and αSMA in lysates from PSC treated with IL11 (10 ng/mL) or IL 6 (10 ng/mL) across the indicated time-points (0 to 24 h). GAPDH serves as a loading control. (**d**) ELISA-based quantification of secreted MMP2 levels in PSC supernatants. (**e**,**f**) Representative immunofluorescence images and quantification of αSMA^+ve^ cells, Collagen I intensity/area and EdU^+ve^ cells at baseline and after 24 h treatment with either recombinant human TGFβ1 (5 ng/mL), IL11 (5 ng/mL), bFGF (10 ng/mL), CTGF (50 ng/mL), PDGF (200 ng/mL) or EDN1 (250 ng/mL). Cells were counterstained with DAPI. Scale bar: 200 µm. (**g**) Matrigel invasion capacity of PSCs was determined at baseline and after 24 h treatment with PDGF (20 ng/mL) or with increasing concentrations of IL11 (5–20 ng/mL). Scale bars: 150 µm. AU: Arbitrary Unit. Data are represented as mean ± SD in panel (**d**) and median and whiskers extending from minimum to maximum values in panels (**f**,**g**). *p* values were determined by one-way ANOVA with Dunnet’s correction. BL: baseline.

PSCs are estimated to comprise approximately 5% of the pancreatic cells [7]. To quantify the expression of Il11 receptor alpha (*Il11ra1*), Il6 receptor alpha (*Il6ra*) and *Il6st* (gp130) in healthy mouse pancreas, we examined the single cell RNA-seq data from the *Tabula muris* Consortium [20]. tSNE plots show high expression of *Il11ra1* in PSCs, as compared to the expression of the alpha receptor for IL6 (*Il6ra*), which was at lower levels (Figure 1a). The common receptor subunit for signal transduction of the *IL6* family of cytokines, gp130, was expressed across a wide range of cell-types in the mouse pancreas. To evaluate the expression of protein levels of IL11RA, IL6RA and gp130 expression in primary human PSCs immunostaining was performed. This revealed that human PSCs express high levels of IL11RA and gp130, while IL6RA is lowly expressed (Figure 1b), recapitulating the expression pattern inferred from murine single cell RNA-seq data. 

Next, to compare the effects of IL11 to those of IL6 in PSCs, we profiled human cells treated with an equivalent dose (10 ng/mL) of either recombinant IL11 or IL6 over a 24 h period by Western blot analysis, and monitored for STAT3 and ERK activation (phosphorylation) along with markers of myofibroblast transformation (αSMA expression and matrix metalloproteinase 2 (MMP2) secretion). IL11 treatment induced a rapid and sustained activation of ERK, which coincided with the progressive increase in αSMA expression and MMP2 secretion at later time points (Figure 1c,d). IL11 induced early and brief activation of STAT3, which then returned to baseline after 2 h of stimulation. In contrast, IL6 stimulation did not activate ERK, but instead potently induced sustained STAT3 phosphorylation. IL6 did not upregulate αSMA or induced MMP2 secretion, showing that it does not induce PSC-to-myofibroblast transformation (Figure 1c,d).

To further assess the effects of IL11 on PSCs, we incubated PSCs with IL11 (24 h) and compared its effects with various other cytokines implicated in pancreatitis, such as transforming growth factor-beta 1 (TGFβ1), basic fibroblast growth factor (bFGF), connective tissue growth factor (CTGF), platelet derived growth factor (PDGF), and endothelin 1 (EDN1). We monitored PSC activation and proliferation by immunofluorescence quantification of αSMA, type I collagen (Collagen I) and EdU incorporation. IL11 significantly upregulated protein markers of myofibroblast transformation (αSMA and Collagen I expression) and increased cell proliferation at levels comparable to the other factors tested (Figure 1e,f). Furthermore, cellular chemotaxis and ECM invasion were induced by IL11 in a dose-dependent manner (Figure 1g).

### 2.2. IL11-Dependent ERK Signaling Is Required for Pancreatic Stellate Cell Activation

**Figure 2:**
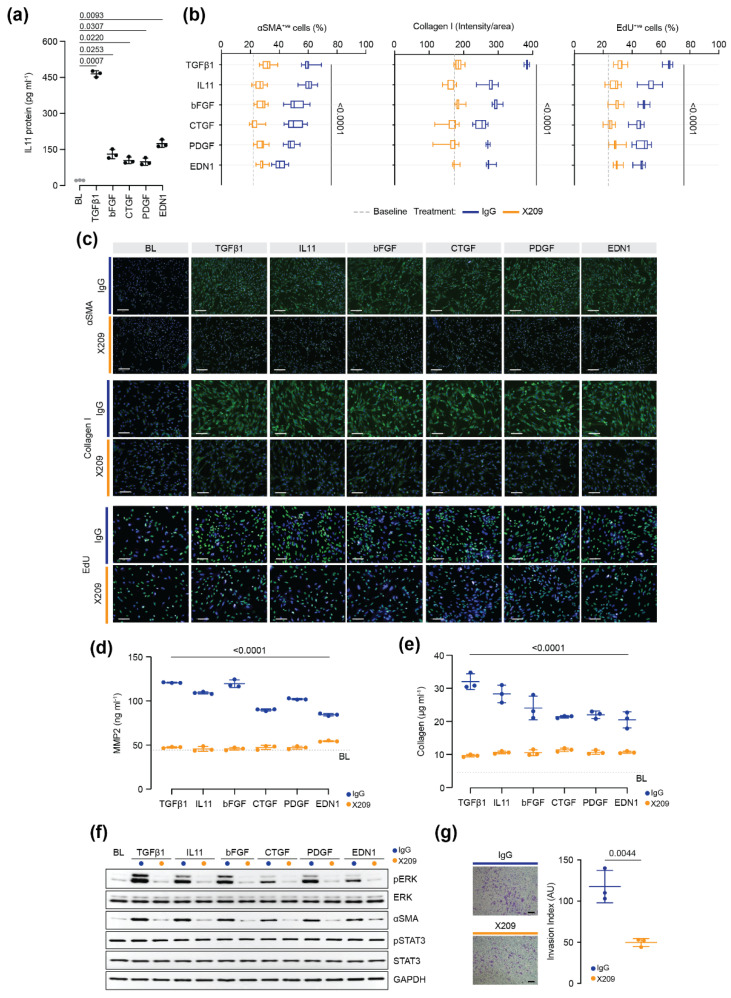
Autocrine IL11 signalling is important downstream of several pancreatitis factors. (**a**) ELISA of secreted IL11 from PSCs after 24 h treatment with recombinant human TGFβ1 (5 ng/mL), bFGF (10 ng/mL), CTGF (10 ng/mL), PDGF (200 ng/mL), EDN1 (250 ng/mL). (**b**,**c**) Representative immunofluorescence images and quantification of αSMA^+ve^ cells and collagen I immunostaining of PSCs treated with IgG or the neutralizing IL11RA antibody (X209, 24 h) and profibrotic cytokines listed in panel (**a**). Cells were counterstained with DAPI. Scale bars: 200 µm. (**d**) ELISA of secreted MMP2 and (**e**) Sirius Red quantification of secreted collagen in the culture supernatant of PSCs treated as depicted in panel (**c**). (**f**) Western blot analysis of phosphorylated and total ERK and STAT3, and αSMA in lysates of PSCs treated with various profibrotic stimuli with either IgG or X209 (2 µg/mL, 24 h). (**g**) Matrigel invasion capacity of PSCs treated with IgG or X209 (2 µg/mL) and PDGF (20 ng/mL). Scale bars: 150 µm. AU: arbitrary unit. Data are represented as mean ± SD in (**a**,**d**,**e**,**g**) or as median and whiskers extending from minimum to maximum values in panel (**b**). *p* values were determined by one-way ANOVA with Dunnett’s correction in panel **a**, two-way ANOVA (Sidak’s correction) in panels (**b**,**d**,**e**) and by Student’s *t* test in (**g**). BL: baseline.

Having observed that IL11 induces PSC activation, we investigated whether autocrine IL11 signalling plays a more general role in PSC activation. First, we performed ELISA-based measurements of IL11 in PSC culture supernatants from cells stimulated with various pathogenic factors and found that these stimuli significantly induced IL11 secretion (Figure 2a). We next tested whether these various stimuli were dependent on IL11 for their profibrotic effects by blocking autocrine IL11 signalling with a monoclonal neutralising IL11RA antibody (X209) [19] Compared to an IgG control antibody, PSCs incubated with X209 did not transform into myofibroblast-like cells and expressed significantly reduced αSMA and collagen I along with reduced cell proliferation after stimulation with pancreatitis factors (Figure 2b,c). X209 also reduced the amount of MMP2 and soluble collagen secreted by PSCs following stimulation (Figure 2d,e). X209 blocked the effects of IL11 itself, as expected. Western blot analysis confirmed that X209 treatment reduced PSC activation downstream of these various profibrotic stimuli via specific inhibition of ERK (Figure 2f). On the other hand, STAT3 phosphorylation, which was not induced by the various factors tested, was unchanged by X209 treatment (Figure 2f). Furthermore, Matrigel assays demonstrated that X209 significantly reduced ECM invasion by PSCs stimulated with PDGF (Figure 2g). These results show that autocrine IL11 activity is a shared downstream fibrogenic effector of multiple cytokines implicated in PSC activation.

**Figure 3:**
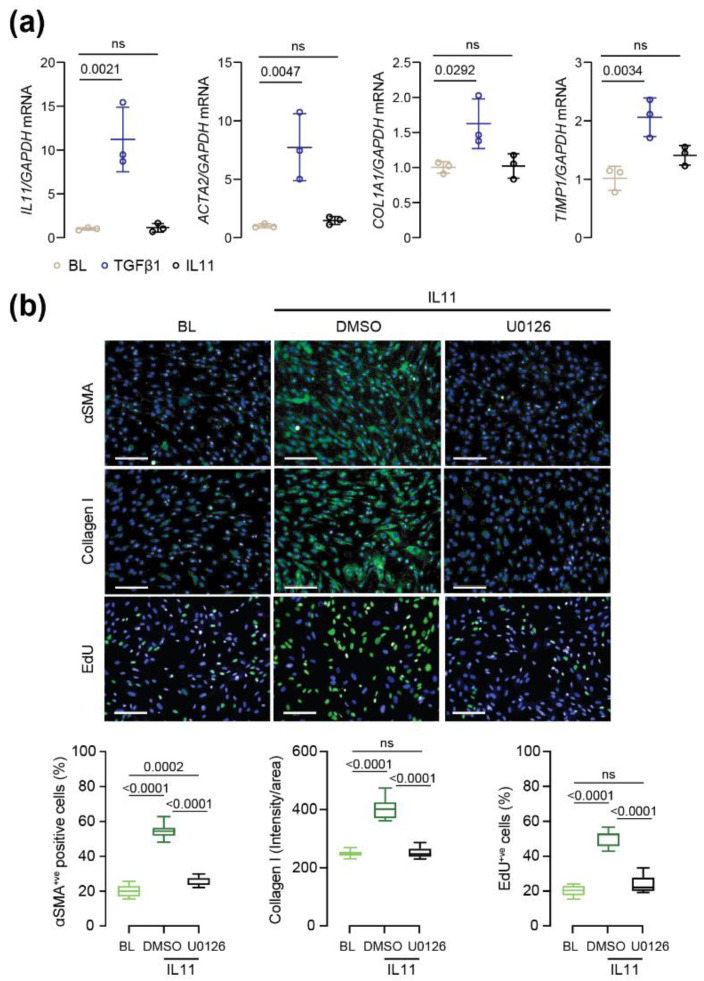
IL11 drives pancreatic stellate cell activation via ERK signalling and post-transcriptional effects. (**a**) RNA expression of *IL11*, *ACTA2*, *COL1A1* and *TIMP1* in PSCs treated with recombinant human TGFβ1 or IL11 (5 ng/mL; 24 h). (**b**) Representative immunofluorescence images and quantification of αSMA^+ve^ cells, Collagen I intensity/area and EdU^+ve^ cells at baseline and after 24 h treatment with IL11 (5 ng/mL) and ERK inhibitor U0126 (10 µM). Data are represented as mean ± SD in panel (**a**) and as median and whiskers extending from minimum to maximum values in panel (**b**). Scale bars: 100 µm. *p* values were determined by one way ANOVA (Dunnet’s correction) in (**a**) and one way ANOVA (Tukey’s correction) in (**b**). BL: baseline.

To gain additional insights into the mechanisms by which IL11 induces PSC activation, we performed RT-qPCR on lysates from PSCs treated with either IL11 or TGFβ1 (24 h). As compared to TGFβ1-treatment, which significantly induced the expression of several fibrotic genes (*ACTA2*, *COL1A1*, *TIMP1* and *IL11*), IL11 treatment did not lead to an upregulation of these genes at the RNA level, as expected, as IL11’s effects are mediated in stroma cells mostly at the level of gene translation (Figure 3a) [21,22]. 

To further examine whether ERK signalling is of specific importance for IL11-induced PSC activation, we stimulated PSCs with IL11 in the presence of the ERK inhibitor U0126. This showed that the profibrotic and proliferative effects of IL11 on PSCs were significantly reduced by ERK inhibition, as measured by immunofluorescence quantification of αSMA, collagen I and EdU incorporation (Figure 3b). 

### 2.3. A Neutralizing IL11RA Antibody Reduces Tissue Damage in Acute Pancreatitis 

**Figure 4:**
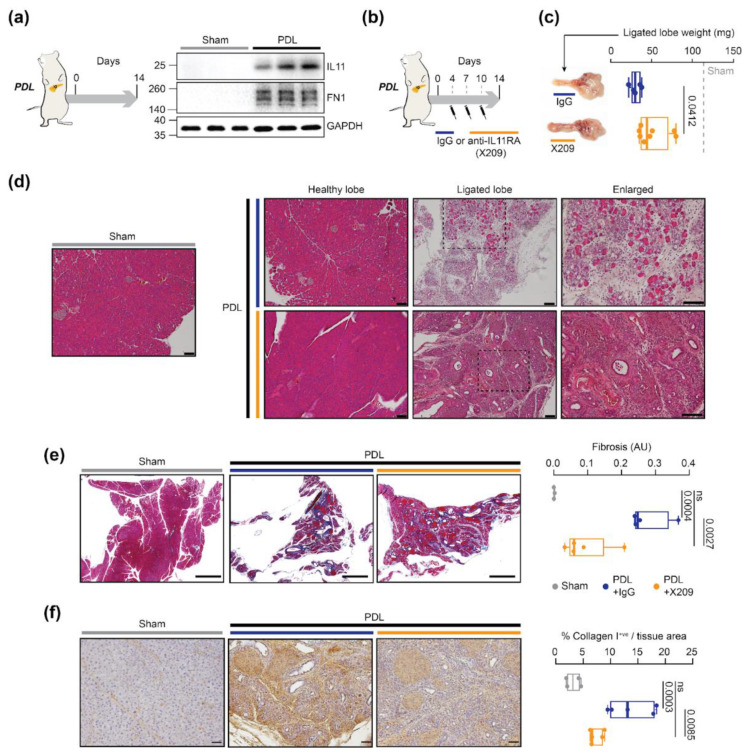
IL11RA antibody treatment reduces pancreatic fibrosis in a mouse model of pancreatitis. (**a**) Schematic of the induction of pancreatitis in wildtype C57BL/6 mice by pancreatic duct ligation (PDL) and Western blot analysis of IL11 and fibronectin (FN1) expression in pancreatic lysates post-sham or 14 days post-PDL surgery (n = 3/group). (**b**) Schematic of the administration timepoints of neutralizing IL11RA antibody (X209) or IgG control antibody treatment in the PDL model. (**c**) Gross pancreas anatomy and the tissue weights of the ligated splenic lobe in X209 or IgG treated mice. (**d**) Hematoxylin and eosin staining of pancreatic sections from the healthy or ligated splenic lobes of X209 or IgG treated mice. Scale bars: 100 µm. (**e**) Masson’s trichrome staining images and collagen quantification of fibrosis in the ligated splenic lobes of X209 or IgG treated mice (n = 3–4). Scale bars: 1000 µm. AU: arbitrary unit. (**f**) Collagen I immunostaining of the ligated splenic lobes of X209 or IgG treated mice. Scale bars: 50 µm. Data shown as median and whiskers extending from minimum to maximum values. *p* values were determined by Student’s *t*-test in (**c**) and one way ANOVA (Tukey’s correction) in (**e**,**f**).

To determine the role of IL11 in PSC activation and pancreatitis in vivo, we used the established pancreatic duct ligation (PDL) mouse model of pancreatitis [23,24]. Wildtype C56BL/6 mice were subjected to PDL and the ligated splenic lobes of the pancreas were collected on day 14, a time point which coincides with robust fibro-inflammation in this model. Western blot analysis of tissue homogenates revealed that IL11 protein expression in the ligated part of the pancreas was significantly upregulated 14 days following PDL, and this was concurrent with increased expression of fibronectin (Figure 4a). 

Next, we tested the therapeutic potential of X209 for the treatment of pancreatitis. X209 was administered by intraperitoneal injection (20 mg/kg body weight) on day 4, 7 and 10 post-PDL (Figure 4b). Mice were euthanized 14 days post-PDL and the ligated splenic lobes of the pancreas were evaluated. By gross morphology analysis, as compared to sham controls, we observed a reduction in tissue weights of the ligated part of the pancreas in IgG-treated animals, indicative of parenchymal necrosis and atrophy. As compared to IgG-treated animals, X209-treated animals had significantly greater ligated lobe weights (Figure 4c). To further characterize pancreatic injury in these mice, we performed hematoxylin and eosin staining of pancreatic sections. As compared to healthy non-ligated pancreas, the ligated splenic lobes of IgG treated mice showed morphological characteristics of severe pancreatitis, with profound loss of acini, immune cell infiltration and replacement of damaged regions with fibrotic tissue (Figure 4d). These histological characteristics were markedly reduced in X209 treated mice as compared to IgG treated controls. Further histology assessment of injured pancreases by Masson’s trichrome staining showed that X209 significantly reduced collagen deposition and fibrosis as compared to IgG-treated mice (Figure 4e). Furthermore, collagen I immunostaining in the pancreas of X209-treated mice was significantly reduced as compared to IgG-treated controls (Figure 4f).

**Figure 5:**
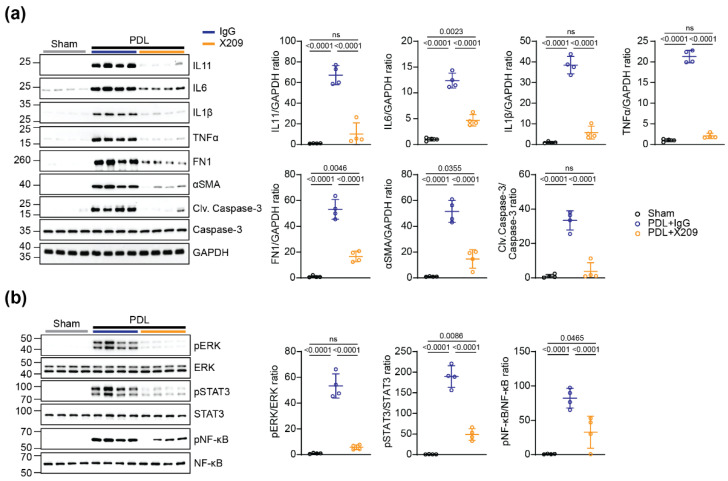
IL11RA antibody treatment attenuates pancreatic inflammation and pathological signalling in a mouse model of pancreatitis. (**a**) Western blot analysis of IL11, IL6, IL1β, TNF, FN1, αSMA, cleaved (Clv.) and total caspase-3 and (**b**) phosphorylated and total protein levels of ERK, STAT3 and NF-kB in lysates from the ligated splenic lobes of X209 or IgG treated mice (n = 4/group). GAPDH served as loading control in (**a**). *p* values were determined by one way ANOVA (Tukey’s correction).

We studied the impact of X209 on fibrosis and inflammation in pancreatitis by Western blot analysis of pancreatic homogenates from IgG and X209-treated mice with PDL. This showed that ECM and myofibroblast proteins (fibronectin and αSMA), inflammation-related cytokines (IL6, IL1β and TNFα) and IL11, which is also pro-inflammatory [25], were strongly upregulated in PDL-challenged mice treated with IgG control antibody. Notably, X209 treatment resulted in considerably lower levels of all these proteins post-PDL challenge (Figure 5a). Furthermore, the activation of caspase-3, an essential mediator of apoptosis, was greatly increased post-PDL but was significantly reduced by X209 (Figure 5a).

To investigate the effects of X209 on downstream signalling pathways important for fibrosis and inflammation, we profiled the activation status of STAT3, ERK and NF-kB by Western blotting of pancreas homogenates from X209/IgG treated mice with PDL. We observed elevated levels of phosphorylated ERK, STAT3 and NF-kB in mice with PDL and receiving IgG as compared to sham controls. Notably, blocking IL11 signalling with X209 reduced ERK, STAT3 and NF-kB phosphorylation as compared to IgG-treated animals (Figure 5b). 

### 2.4. IL11 and IL11RA Are Elevated Chronic Pancreatitis in Humans

**Figure 6:**
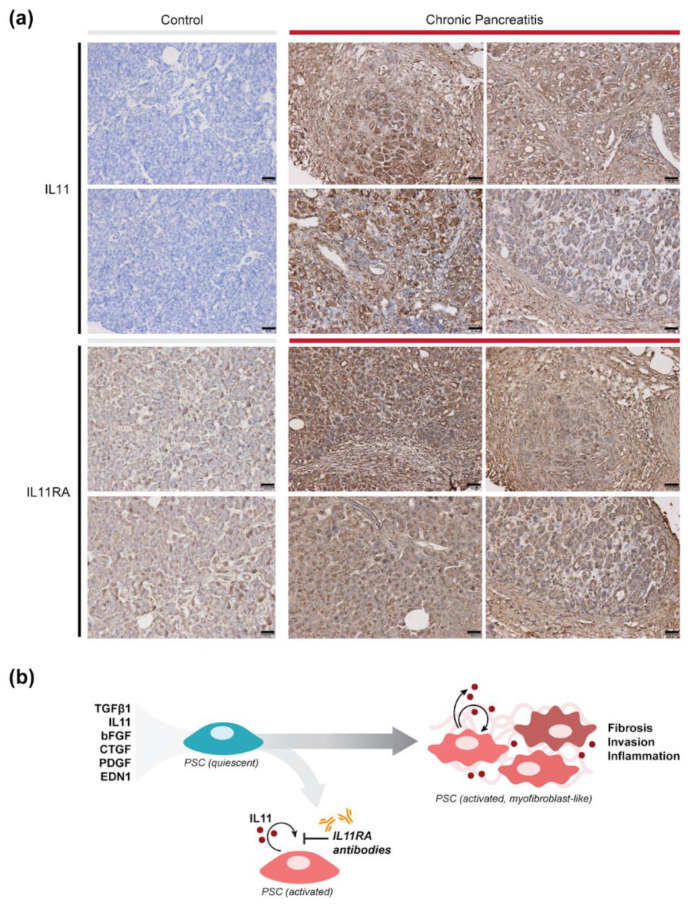
IL11 and IL11RA expression are elevated in human chronic pancreatitis. (**a**) Immunohistochemistry staining of IL11 and IL11RA in normal human (control) or chronic pancreatitis tissue. Scale bars: 50 µm. (**b**) Schematic of the working model of IL11-mediated PSC activation in pancreatitis. Our data suggest that IL11 is induced downstream of multiple pancreatitis factors and acts in an autocrine manner to promote myofibroblast differentiation and its associated effector functions including extracellular matrix production, invasion and the secretion of pro-inflammatory cytokines. Blocking the IL11-autocrine loop using neutralizing IL11RA antibodies prevents myofibroblast differentiation and has protective effects against fibro-inflammation in the pancreas.

We investigated the expression of IL11 and IL11RA in human pancreatitis by performing immunostaining for IL11 and IL11RA in pancreatic tissue sections from patients with chronic pancreatitis and from a control individual. IL11 was not detectable in control pancreases, but was readily detected in pancreatic sections from patients with chronic pancreatitis (Figure 6a). In pancreatitis, IL11 expression was highest in cells within regions of interlobular fibrosis and in acinar cells. Immunostaining for IL11RA revealed that the receptor expression, weakly detected in control pancreas, was elevated in disease (Figure 6a). As such, both IL11 and its receptor are upregulated in the pancreases of patients with chronic pancreatitis.

## 3. Discussion

PSC activation is increasingly recognised as important in pancreatitis, with a number of signalling pathways believed to be involved, including ERK [26]. We show here that IL11 stimulates PSC-to-myofibroblast transformation and that is associated with, and requires, sustained ERK activation. These effects are consistent with our previous studies and support a primarily post-transcriptional role of IL11 for stromal cell activation [19,21,22], although we highlight that IL11 effects on transcription in epithelial cells is described. Our data reveal that multiple pancreatitis factors induce IL11 secretion from PSCs and that autocrine IL11 activity is important for PSC activation, which suggests a non-redundant role for IL11 in pancreatitis (Figure 6b).

Our studies here in primary human PSCs highlight again the distinct signalling differences between IL11 (transient STAT3 activation; sustained ERK activation) and IL6 (sustained STAT3 activation; no ERK activation). Of note, our study did not identify a STAT3-dependent profibrotic role for IL6 in PSCs as was recently proposed by Zheng et al. [14]. A critical difference being that the study by Zheng et al. used an RSV enhancer-driven SV40 T antigen immortalised PSC-like cell line (HP-1) [27]. We have shown that high passage primary cells and immortalised cell lines have different/artefactual signalling and phenotypes, as compared to primary cells, when stimulated with IL6 family members [28]. We suggest that the data shown by Zheng et al. are relevant to transformed HP-1 cells but not primary human PSCs. Instead, in primary human PSCs, we show that IL6 activates STAT3 but does not induce myofibroblast transformation. 

In therapeutic studies, we examined the potential of a neutralising IL11RA antibody to treat pancreatitis. Several rodent studies have shown protective effects of ERK inhibition in experimental acute pancreatitis [29,30], whereas the role for STAT3 activation is less clear [31]. Here, we found that ERK and STAT3 are both activated following PDL and associated with pancreatic fibrosis and inflammation. Blocking IL11 signalling reduced fibrosis and pathological ERK activation as well as inhibiting STAT3 phosphorylation. Taking into account both the in vitro and in vivo findings, we suggest that anti-IL11 therapy in vivo targets PSC activation via specific reductions in ERK activity, but also secondarily inhibits STAT3 in local and infiltrating immune cells, as seen previously in the lung [25].

In addition to the profibrotic effects of IL11, recent data have shown that IL11 signalling plays an important role in stromal-driven inflammation in the lung, colon and liver [19,25,32,33]. Although we did not investigate the specific role of IL11 in the immune response in pancreatitis, we found that blocking IL11 signalling had anti-inflammatory effects and reduced IL6, IL1β and TNFα protein levels as well as diminishing NF-κB and STAT3 activation. In contrast to our findings, an early study showed that administration of recombinant human IL11 reduced disease severity in the caerulein-induced mouse model of pancreatitis [18]. We recently found that recombinant human IL11 is paradoxically cytoprotective in mice due to its competitive inhibition of endogenous and pathogenic mouse IL11 activity [34]. This may explain the discrepancy. 

Our analysis of human pancreatic tissues, although limited, revealed that IL11 and IL11RA are increased in chronic pancreatitis. These findings are consistent with an earlier study that showed elevated IL11 levels in the serum of patients with pancreatitis [16]. We suggest that the elevated levels of IL11 in acinar cells in chronic pancreatitis could be linked with additional effects of IL11 in the pancreas, outside of the stroma. Indeed, there are emerging links between IL11 and epithelial cell dysfunction, notably epithelial-to-mesenchymal transition, in other organs such as the liver, kidney and lung [35,36,37,38]. We end by suggesting that therapies targeting IL11 signalling may be considered as a therapeutic approach for patients with pancreatitis.

## 4. Materials and Methods

### 4.1. Recombinant Proteins

Commercial recombinant proteins: human TGFβ1 (PHP143B; Bio-Rad, Oxford, UK), human basic fibroblast growth factor (bFGF, 233-FB-025; R&D Systems, Minneapolis, MN, USA), human connective tissue growth factor (CTGF, PHG0286, ThermoFisher Scientific, Santa Clara, CA, USA), human PDGF (220-BB-010; R&D Systems, Minneapolis, MN, USA), human endothelin 1 (EDN1, 1160, Tocris, Bristol, UK), and human IL-6 (206-IL-010; R&D Systems, Minneapolis, MN, USA). Custom recombinant protein: human IL11 (UniProtKB:P20809, Genscript, Piscataway Township, NJ, USA).

### 4.2. Chemicals

Bovine serum albumin (BSA, 7906, Sigma-Aldrich, St. Louis, MO, USA), DAPI (D1306, ThermoFisher Scientific, Santa Clara, CA, USA), 16% Formaldehyde (*w/v*), Methanol-free (Cat #28908, Pierce™ ThermoFisher Scientific, Santa Clara, CA, USA), 10% Neutral Buffered Formalin (3800598, Leica), Triton-X 100 (T8787, Sigma-Aldrich, St. Louis, MO, USA), Tween-20 (170-6531, Bio-Rad), and U0126 (#9903, Cell Signaling Technology, Danvers, MA, USA).

### 4.3. Cell Culture

Primary human pancreatic stellate cells (PSCs; ScienCell, Carlsbad, CA, USA; Cat #3830, Lot #14289) were isolated from human pancreas healthy samples and were maintained in Stellate cell medium (SteCM, Cat #5301, ScienCell, Carlsbad, CA, USA) supplemented with stellate cell growth supplement(ScienCell, Carlsbad, CA, USA Cat #5352), 2% fetal bovine serum (ScienCell, Carlsbad, CA, USA; Cat #0010) and 1% Penicillin-streptomycin (ScienCell, Carlsbad, CA, USA Cat #0503), in an incubator at 37 °C and with an atmosphere of 5% CO_2_. Unless otherwise specified, PSCs were serum-starved overnight prior to 24 h stimulation with different doses of various recombinant proteins, as outlined in the main text and/or figure legends. All experiments were carried out at low cell passage (<P3).

### 4.4. Immunofluorescence Staining

PSCs were seeded in 8-well chamber slides (1.5 × 10^4^ cells/well). Cells were washed with PBS twice and fixed in 4% paraformaldehyde (PFA) for 20 min. Non-specific sites were blocked with 5% BSA in PBS for 2 h. Cells were incubated overnight (4 °C) with IL11RA, IL6RA or gp130 antibodies (see Table 1 for details) diluted in blocking solution, followed by incubation with the appropriate secondary antibodies for one hour at room temperature. Negative control cells were only stained with the secondary antibodies. Chamber slides were dried in the dark and 5 drops of mounting medium with DAPI (D1306, Thermo Fisher Scientific, Santa Clara, CA, USA) were added to the slides for 15 min prior to imaging by fluorescence microscope (Leica).

### 4.5. High-Content Imaging Assays

The PSC staining procedure for high-content imaging assays was performed as described previously [27]. EdU-AlexaFluor488 was incorporated using a Click-iT EdU labelling kit (C10350, ThermoFisher Scientific, Santa Clara, CA, USA) according to the manufacturer’s protocol. Primary antibodies against αSMA and collagen I (refer to Table 1 for details) were diluted in blocking solution (0.5% BSA and 0.1% Tween-20 in PBS) and incubated overnight, followed by incubation with the appropriate Alexa Fluor 488 secondary antibodies for one hour at room temperature. Cells were counterstained with 1 µg/mL DAPI (D1306, Thermo Fisher, CA, USA) in blocking solution. Two wells per condition were imaged at a minimum of 7 fields/well, using Operetta high-content imaging system 1483 (PerkinElmer). The quantification of αSMA^+ve^ and EdU^+ve^ cells was performed using Harmony software version 3.5.2 (PerkinElmer, Waltham, MA, USA). The measurement of collagen I fluorescence intensity per area (normalized to the number of cells) was performed with Columbus 2.9.0 (PerkinElmer, MA, USA).

### 4.6. ELISA and Sirius Red Collagen Assay

Secreted levels of IL11 and MMP-2 in the culture supernatant of PSC were quantified using the human IL11 and MMP2 Quantikine ELISA kit (D1100; MMP200, R&D systems, MN, USA) according to manufacturer’s instructions. For quantification of secreted collagen by PSC, the supernatant was first concentrated using polyethylene glycol solution (90626 Chondrex, Woodinville, WA, USA) and quantified using the Sirius Red total collagen detection kit (9062, Chondrex, WA, USA) according to the manufacturer’s protocol. 

### 4.7. RT-qPCR

Total RNA was isolated from PSCs using Trizol (15596026, Thermo Fisher Scientific, Santa Clara, CA, USA) followed by purification using the RNeasy Mini Kit (74104, Qiagen, MD, USA) and the cDNA was prepared using iScript cDNA synthesis kit (1708891, Bio-Rad) following the manufacturer’s protocols. RT-qPCR was performed with QuantiFast SYBR Green PCR kit (204054, Qiagen, MD, USA) using Applied Biosystem StepOnePlus Real-Time PCR System (4376600, ThermoFisher Scientific, Santa Clara, CA, USA). RNA expression was normalized to GAPDH expression using the 2^−ΔΔCt^ method to calculate fold change. Primer sequences are listed in Table 2. 

### 4.8. Matrigel Invasion Assay

The invasion capacity of PSC was assayed using 24-well Boyden chamber invasion assays (CBA-110, Cell Biolabs Inc., San Diego, CA, USA). PSC were serum starved in basal stellate cell medium before invasion assays. Equal numbers of PSCs were then seeded in duplicates onto the apical chambers containing ECM-coated Matrigel. PSCs were allowed to invade towards basal Stellate Cell Medium containing chemoattractant PDGF (20 ng/mL) or increasing concentrations of IL11 (0 to 20 ng/mL). The cells that invaded the Matrigel matrix towards the bottom chamber were stained with cell staining solution (Cell Biolabs Inc.) and 5 non-overlapping fields (40× magnification) of each membrane were imaged and cells were counted. For antibody experiments, PSC were incubated with 2 µg/mL of IgG or X209 antibodies for 15 min prior to adding chemoattractants.

### 4.9. Pancreatic Duct Ligation (PDL) Model

C57BL/6 mice were purchased from InVivos (Singapore). Mice were maintained in a specific pathogen-free environment and given ad libitum access to food and water. Pancreatitis was induced in 10–12-week-old mice by dissection and ligation of the main splenic pancreatic duct at the junction between the gastric and the duodenal lobe. Caution was taken to avoid ligation of the bile duct and the adjacent artery. Mice received intraperitoneal injections of IgG (11E10, Aldevron, Freiburg im Breisgau, Germany) or neutralizing IL11RA antibody (X209, Aldevron, Freiburg im Breisgau, Germany) at a dose of 20 mg/kg body weight on days 4, 7 and 10 post ligation and the mice were sacrificed on day 14. 

### 4.10. Histology and Immunohistochemistry

Pancreatic tissue from mice that underwent PDL was fixed in 10% neutral buffered formalin for 24 h, embedded in paraffin and sectioned for Masson’s trichrome staining. For immunohistochemistry, pancreatic tissue sections were incubated overnight with primary antibodies (anti-COL1A1, ab21286, Abcam, Cambridge, UK) for mouse tissues, or with either anti-IL11 antibody (X203, Aldevron, Freiburg im Breisgau, Germany) or anti-IL11RA (X209, Aldevron, Freiburg im Breisgau, Germany) for human pancreatic tissue, and visualized using ImmPRESS HRP IgG polymer detection kit (Vector Laboratories, CA, USA). Normal and pancreatitis human tissue sections were obtained from Novus Biologicals, CO, USA (NBP2-30191) and Biomax Inc., Rockville, MD, USA (Catalog: BIC14011b), respectively. Quantification of Masson’s trichrome staining and immunohistochemistry was performed using ImageJ Fiji (version 1.50) using colour deconvolution using the Masson’s trichrome or H DAB analysis options, respectively. Threshold adjustments were subsequently performed relative to sham control images, and the positively stained area was then expressed relative to the tissue area.

### 4.11. Immunoblotting

Pancreatic tissue samples were homogenized in RIPA Buffer (89901, Thermo Scientific, CA, USA) containing protease and phosphatase inhibitors (Roche). Protein lysates were resolved by SDS-PAGE and transferred onto PVDF membranes. Unspecific sites were blocked in TBS-T supplemented with 5% non-fat dry milk. Primary antibodies for Western blots (listed in Table 1) were diluted in 1% non-fat dry milk and the membranes were incubated in primary antibody solutions overnight. After incubation with the appropriate HRP-conjugated secondary antibody, bands were visualized using the ECL detection system (Pierce).

### 4.12. Statistical Analysis

Statistical analyses were performed using GraphPad Prism software (version 9). Two-tailed Student’s *t*-test was used for experimental setups requiring testing of two conditions solely. Otherwise, one-way ANOVA was performed and *p*-values adjusted according to Dunnett’s (several experimental groups compared to one condition) or Tukey’s (two treatment groups compared to each other across conditions) multiple testing correction or two-way ANOVA with Sidak’s correction. The criterion for statistical significance was set at *p* < 0.05.

## Figures and Tables

**Table 1:** Antibodies used in this study.

Antibodies	Source	Catalogue	Usage (Dilution)
Anti-α-Smooth Muscle Actin	Abcam	ab7817	Immunofluorescence (1:500)
Anti-Collagen I	Abcam	ab34710	Immunofluorescence (1:500)
Anti-Collagen I	Abcam	ab21286	Immunohistochemistry (1:100)
Anti-gp130	Thermo Fisher	PA5-99526	Immunofluorescence (1:100)
Anti-IL6RA	Thermo Fisher	MA1-80456	Immunofluorescence (1:100)
Anti-IL11RA	Abcam	ab125015	Immunofluorescence (1:100)
Anti-IL11RA	Aldevron	X209	Immunohistochemistry (1:100)
Anti-rabbit Alexa Fluor 488 secondary antibody	Abcam	ab15077	Immunofluorescence (1:1000)
Anti-mouse Alexa Fluor 488 secondary antibody	Abcam	ab1510113	Immunofluorescence (1:1000)
Anti-IL11	Aldevron [39]	X203	Western blot (1:5000); Immunohistochemistry (1:500)
Anti-IL6	Cell Signaling Technology	12912	Western blot (1:1000)
Anti-IL1β	Cell Signaling Technology	12242	Western blot (1:1000)
Anti-TNFα	Cell Signaling Technology	11948	Western blot (1:1000)
Anti-pERK1/2	Cell Signaling Technology	4370	Western blot (1:1000)
Anti-ERK1/2	Cell Signaling Technology	4695	Western blot (1:1000)
Anti-pSTAT3	Cell Signaling Technology	4113	Western blot (1:1000)
Anti-STAT3	Cell Signaling Technology	4904	Western blot (1:1000)
Anti-pNF-κB	Cell Signaling Technology	3033	Western blot (1:1000)
Anti-NF-κB	Cell Signaling Technology	8242	Western blot (1:1000)
Anti-Cleaved Caspase-3	Cell Signaling Technology	9664	Western blot (1:1000)
Anti-Caspase-3	Cell Signaling Technology	9662	Western blot (1:1000)
Anti-α-Smooth Muscle Actin	Cell Signaling Technology	19245	Western blot (1:1000)
Anti-GAPDH	Cell Signaling Technology	2118	Western blot (1:1000)
Anti-Fibronectin	Abcam	ab2413	Western blot (1:1000)
Anti-rabbit IgG, HRP-linked antibody	Cell Signaling Technology	7074	Western blot (1:2000)
Anti-mouse IgG, HRP-linked antibody	Cell Signaling Technology	7076	Western blot (1:2000)

**Table 2:** Primer sequences for RT-qPCR.

Host	Gene	Forward Primer	Reverse Primer
Human	*ACTA2*	5′-CTGTTGTAGGTGGTTTCATGGA-3′	5′-AGAGTTACGAGTTGCCTGATG-3′
Human	*COL1A1*	5′-GAGGGCCAAGACGAAGACATC-3′	5′-CAGATCACGTCATCGCACAAC-3′
Human	*IL11*	5′-GCAGCGGACAGGGAAGGGTT-3′	5′-CCACAGGCTCAGCACGACCA-3′
Human	*TIMP1*	5′-GTGGCACTCATTGCTTGTGG-3′	5′-CAAGGTGACGGGACTGGAAG-3′
Human	*GAPDH*	5′-ACAACTTTGGTATCGTGGAAGG-3′	5′-GCCATCACGCCACAGTTTC-3′

## Data Availability

All data generated in the study are presented in the manuscript.
